# Immunomodulatory Potential of Differently-Terminated Ultra-Small Silicon Carbide Nanoparticles

**DOI:** 10.3390/nano10030573

**Published:** 2020-03-22

**Authors:** Tereza Bělinová, Iva Machová, David Beke, Anna Fučíková, Adam Gali, Zuzana Humlová, Jan Valenta, Marie Hubálek Kalbáčová

**Affiliations:** 1 Biomedical Center, Faculty of Medicine in Pilsen, Charles University, 323 00 Pilsen, Czech Republic; 2 Wigner Research Centre for Physics, 1121 Budapest, Hungary; beke.david@wigner.mta.hu (D.B.); gali.adam@wigner.mta.hu (A.G.); 3 Department of Atomic Physics, Budapest University of Technology and Economics, 1111 Budapest, Hungary; 4 Department of Chemical Physics and Optics, Faculty of Mathematics and Physics, Charles University, 121 16 Prague, Czech Republic; anna.fucikova@email.cz (A.F.); jan.valenta@mff.cuni.cz (J.V.); 5 Institute of Pathological Physiology, 1st Faculty of Medicine, Charles University, 128 53 Prague, Czech Republic; zuzana.humlova@lf1.cuni.cz

**Keywords:** nanoparticles, silicon carbide, cytotoxicity, immune cells, osteoblasts

## Abstract

Ultra-small nanoparticles with sizes comparable to those of pores in the cellular membrane possess significant potential for application in the field of biomedicine. Silicon carbide ultra-small nanoparticles with varying surface termination were tested for the biological system represented by different human cells (using a human osteoblastic cell line as the reference system and a monocyte/macrophage cell line as immune cells). The three tested nanoparticle surface terminations resulted in the observation of different effects on cell metabolic activity. These effects were mostly noticeable in cases of monocytic cells, where each type of particle caused a completely different response (‘as-prepared’ particles, i.e., were highly cytotoxic, –OH terminated particles slightly increased the metabolic activity, while –NH_2_ terminated particles caused an almost doubled metabolic activity) after 24 h of incubation. Subsequently, the release of cytokines from such treated monocytes and their differentiation into activated cells was determined. The results revealed the potential modulation of immune cell behavior following stimulation with particular ultra-small nanoparticles, thus opening up new fields for novel silicon carbide nanoparticle biomedical applications.

## 1. Introduction

The knowledge of nanoparticles (NPs) and their potential application in the field of biomedicine as drug delivery [1,2], therapeutic [3,4,5], and diagnostic platforms and imaging devices [6] have improved significantly over recent years. While particles of different materials and sizes have been studied in detail, only a limited numbers of studies on ultra-small NPs, with sizes of less than 5 nm, have been reported [7,8,9]. Ultra-small silicon carbide NPs, the study of which remains exceptional, are particularly interesting not only due to their biocompatibility and biodegradability [10,11,12] but also due to their extremely small size (approximately 4 nm). The potential use of such particles in biomedicine is, however, only partially understood; only a small number of isolated studies taking advantage of their natural fluorescence have been conducted to date [11,13,14]. Moreover, the differing surface termination of otherwise identical NPs is of particular interest due to the potential for further modification and cluster formation as well as in terms of their influence on the overall cellular response [15,16,17,18].

As soon as any NP is administered into a biological fluid (a cultivation medium in vitro or blood in vivo), the particles form a new identity from the various components of the surrounding environment that is widely known as the biomolecular or protein corona (PC) since it is formed by biomolecules and, principally, by proteins [19]. This new identity then serves as a particle-cell interaction mediator [20,21,22]. While in in vivo experiments the PC is present constantly and changes over time, the interaction of NPs with cells in vitro can also be tested without the PC (incubation in the medium without fetal bovine serum (FBS) supplementation) which may assist in the understanding of the impact of the NP material itself. Naturally, the commonly presented PC concept cannot be applied in the case of ultra-small NPs since they are mostly of the same size or smaller than the individual components (proteins) that usually form the corona; however, their interaction with FBS proteins should not be overlooked [23].

Any application conducted in vivo should be preceded by a complex study of NP–cell interaction in vitro that must not only take into account the response of tissue-specific cells but also include a broader view of the future administration of the particles into living organisms (e.g., ingestion, inhalation, or intravenous application). Regardless of the administration method, immune cells are likely to be the first type of cells to respond to the presence of NPs in the body since their purpose is to find and react with foreign agents. The method used to introduce NPs into the living organism determines the types of immune cells that respond—monocytes (cells freely circulating in the blood), dendritic cells (cells mostly found in diverse epithelia), or macrophages (tissue-specific cells). Numerous studies have been conducted regarding the influence of various NPs on the immune cell metabolism, the mitochondrial state, and overall viability [24,25], as well as their response to allergic and inflammatory reactions [26,27,28]. Such data is able to assist in both revealing the potential cytotoxicity of NPs and elucidating the mechanisms via which NPs influence cells. Although NPs are viewed primarily as simple carriers, recent studies have reported their immunomodulating properties [5,29,30,31]. 

Herein, three size-identical ultra-small silicon carbide (SiC-based) NPs varying only in their surface termination—NPs as prepared (SiC–x), NPs with NH_2_ groups (SiC–NH_2_) and NPs with OH groups (SiC–OH) on their surface—were studied in the scope of the biological environment. These NPs are shown to have a different impact on various cell types (osteoblasts, monocytes, and macrophages). Influence on the cellular metabolic activity as well as on the state of mitochondria and production of cytokines in short term cultivation is shown with respect to immune cells, indicating the possibilities for further applications of such NPs and showing the crucial importance of surface termination of such NPs. 

## 2. Materials and Methods

### 2.1. Nanoparticle Preparation and Characterization

SiC nanocrystals were prepared via the wet chemical etching method, as reported previously [32,33]. The particles were then terminated with a variety of oxygen-containing species with a high concentration of carboxyl groups [33]. The preparation of the OH-terminated nanoparticles (SiC–OH) involved the reduction of SiC–x NPs via NaBH_4_ in an aqueous solution [34]. The SiC–OH were then silanized for the preparation of the SiC–NH_2_ particles. Briefly, after changing the solvent from deionized water to ethanol with a 1kDa Pall Macrosep ( Pall Co., New York, NY, USA) filter, 10 µL/mL of (APDMES, Sigma-Aldrich, St. Louis, MO, USA) was added to the solution at 40 °C for 2 h. The excess reactant was removed, and the solvent was changed back to DI water with a 1kDa Pall Macrosep filter. Size distribution of NPs was measured by means of atomic force microscopy (AFM, Bruker Dimension Icon atomic force microscope using Bruker (Bruker, Palaiseau, France) Tapping mode and Bruker MPP-11100-10 probe (Bruker, Palaiseau, France), Pall Macrosep). Fourier transform infrared spectra (FTIR) was measured using Bruker Tensor37 (Bruker Co., Billerica, MA, USA) spectrometer equipped with a zinc selenide horizontal multi bounce attenuated total internal reflection accessory, and nuclear magnetic resonance spectra (NMR) was measured using Varian NMR System (Varian, Palo Alto, CA, USA), 600 MHz.

### 2.2. Zeta Potential Determination

The zeta potential (ζ) of the SiC-based NPs was determined in water (standard solution), DMEM and RPMI 1640 media as well as in both the media with the addition of 5% FBS. The differing NPs in the same amount were pre-incubated overnight in the respective solutions under constant rotation at room temperature (RT). The NPs were centrifuged at 13,000 g for 30 min at RT. The pellet was washed in phosphate buffer saline (PBS) and centrifuged; the process was repeated six times. The final pellet was resuspended in 100 µL of PBS and subsequently diluted with distilled water to achieve a maximal conductivity of 1.5 mS/cm and measured immediately. The ζ was measured using a Zetasizer Nano Series and software Malvern Zetasizer software ver. 7.13 (Malvern Pananalytical Ltd., Malvern, UK). 

### 2.3. Cell Culture

The human osteoblastic cell line (SAOS-2, DSMZ, Braunschweig, Germany) was cultured in McCoy’s 5 A medium (GE Healthcare – HyClone, Sigma-Aldrich, St. Louis, MO, USA) supplemented with 15% FBS (Biosera, Nuaille, France), L-glutamine (Life Technologies, Thermo Fischer Scientific, Waltham, MA, USA), 10,000 U/mL of penicillin and 10 μg/mL of streptomycin (both Sigma-Aldrich, St. Louis, MO, USA) in a CO_2_ humidified incubator at 37 °C. The human monocytic cell line (suspension, THP-1, ATCC) was cultured in RPMI 1640 medium (Biowest, Nuaillé, France) supplemented with 10% FBS (Biosera, Nuaille, France), L-glutamine (Life Technologies, Thermo Fischer Scientific, Waltham, MA, USA), 10,000 U/mL of penicillin and 10 μg/mL of streptomycin (both Sigma-Aldrich, St. Louis, MO, USA). The differentiation of the suspension monocytic THP-1 cells into adherent macrophage-like cells was performed via the following method. The cells were seeded at a concentration of 160,000 cells/cm^2^ on 6-well plate and cultivated for 72 h in a standard cultivation medium supplemented with 1 µM of phorbol 12-myristate13- acetate (PMA, Sigma-Aldrich, St. Louis, MO, USA). The adherent THP-1 were then trypsinized and used for further experiments. The differentiation of the suspension monocytes into dendritic-like cells was induced via the cultivation of the cells in 2 ml of standard cultivation medium supplemented with 0.1 µg/mL of GM-CSF and 0.1 µg/mL of IL-4 (both Thermo Fischer Scientific, Waltham, MA, USA) for 72 h, whereupon 1 ml of fresh standard cultivation medium supplemented with GM-CSF and IL-4 was added followed by cultivation for further 48 h (5 days of treatment in total).

### 2.4. Metabolic Activity Determination

The adherent cells were pre-seeded in the respective standard cultivation medium at a concentration of 10,000 cells/cm^2^ in 96-well plates for 24 h. The cells were then washed with pre-warmed PBS and the NPs (25, 50, and 100 µg/mL) in the corresponding medium (DMEM for SAOS-2 and RPMI 1640 for adherent THP-1), supplemented with 5% FBS or non-FBS-supplemented, were added to the cells; corresponding blanks were also prepared (only the medium with the NPs). After 6 h, the medium with the NPs was replaced with the corresponding NP-free medium supplemented with 5% FBS and cultivation continued up to 24 h. The medium was then discarded and fresh medium with 10% MTS solution was added to the wells. MTS colorimetric assay (Cell Titer961® Aqueous One, Promega, Madison, WI, USA) was used for metabolic activity testing purposes. The optical density was measured (Labsystem Multiskan MS, Vantaa, Finland) at 492 nm and 620 nm as the reference. The corresponding blanks were subtracted and the data was presented as a percentage of the control values (untreated cells).

In the case of the THP-1 suspension (monocytes), the cells were seeded on 96-well U bottom plate at a concentration of 10,000 cells/cm^2^ in 90 µl of medium supplemented with 5% FBS and with no FBS supplementation. Subsequently, 10 μl of NPs diluted in the corresponding medium were added so as to attain final concentrations of 25, 50, and 100 µg/mL. The cells were incubated in a humidified CO_2_ incubator at 37 °C on a shaker. After 6 h, the appropriate amount of FBS was added to the non-supplemented wells (non-standard conditions) so as to attain a final concentration of 5% FBS, and the shaker was switched off. With respect to the MTS measurement, the plates were centrifuged (210 g, 5 min, RT) and 50 µl of the supernatant was aspirated and stored in a freezer for further processing (cytokine detection assay). 50 µl of 20% MTS solution in 5% RPMI was then added so as to attain a final concentration of 10% MTS in the sample. The cells were slightly resuspended and incubated for 2 h in the humidified CO_2_ incubator at 37 °C. The optical density was measured as described above. 

### 2.5. Flow Cytometry Determination of the Cell Number, Mitochondrial Mass, and Potential

The suspension THP-1 cells were cultured and treated as described above with a volume of 250 μl (8,250 cells/well) for the cell number analysis. The 96-well U bottom plate was transferred after the desired incubation time to a flow cytometer (BD FACS Canto, Franklin Lakes, NJ, USA) and the total cell count in a volume of 50 µl was calculated.

The cells were cultured in 6-well plates at a concentration of 10,000 cells/cm^2^ (without FBS supplementation) with 100 µg/mL of NPs for the detection of the mitochondrial mass and potential. The cells were incubated in the humidified CO_2_ incubator at 37 °C on a shaker. After 6 h, the appropriate amount of FBS was added so as to attain a final concentration of 5% FBS, and the shaker was switched off; cultivation continued up to 24 h. The cells were then transferred to FACS tubes and stained using MitoTracker™ probes. The mitochondrial mass was detected using MitoTracker™ Green FM (M7514, Invitrogen, Carlsbad, CA, USA) and the mitochondrial potential using MitoTracker™ Red FM (M22425, Invitrogen, Carlsbad, CA, USA). The incubation time of the cells with both probes was 30 min in the humidified CO_2_ incubator. The positive control (cells with an uncoupled mitochondrial membrane potential) was first treated with 1 mM of carbonyl cyanide-4-(trifluoromethoxy)phenylhydrazone (FCCP, Sigma Aldrich, St. Louis, MO, USA) in the cultivation medium for 30 min in the humidified CO_2_ incubator at 37 °C and then stained employing the aforementioned procedure. The stained samples were analyzed using flow cytometry (BD FACS Canto, 20,000 cells per sample). The data obtained were processed via FlowJo software (FlowJo LLC, version 10.6.1, Franklin Lakes, NJ, USA ). 

### 2.6. Histological Staining for the Morphological Analysis

The suspension THP-1 cells were seeded at a concentration of 10,000 cells/cm^2^ in 6-well dishes in RPMI medium with 5% FBS containing 100 μg/mL of particles. The cells were cultured for 4 days, whereupon 0.5 ml of RPMI medium with 50% FBS was added so as to provide sufficient nutrients. The cultivation proceeded for other 3 days. Then the cells remaining in the suspension were spun (300g, 10 min, RT) and the adherent cells harvested using a standard procedure. All the acquired cells were mixed together, spun (300g, 10 min, RT) and resuspended in PBS. An approximately 10 μl drop of cell suspension was smeared on a glass slide and allowed to dry. The slides were then fixed in methanol (5 min, RT) and stained employing May–Gründwald–Giemsa–Romanowski staining. The slides were analyzed by means of light microscopy (Olympus IX71, Tokio, Japan) with a color camera (Olympus DP74, 100x UPlanPI objective with oil immersion, Tokio, Japan).

### 2.7. Cytokine Detection

Supernatants from the metabolic activity tests were used for cytokine detection purposes. The positive control (monocytes stimulated to inflammation) involved the treatment of the cells with lipopolysaccharide (LPS, 50 µg/mL, E. Coli O111:B4, Sigma-Aldrich, St. Louis, MO, USA) in the same manner as the NP-treated cells. A Human Cytokine Antibody Array (ab133997, Abcam, Cambridge, United Kingdom) was used according to the manufacturer’s manual. The chemiluminescence data was acquired using the ChemiDoc MP (BioRad) system and the data was processed using ImageLab software (BioRad, version 6.0.1., Hercules, CA, USA). The data were normalized and expressed as a percentage of the untreated control.

### 2.8. Statistical Analysis

All the data were presented in the form of the means of all the experimental values related to the control from three biologically independent experiments running in duplicate (at least) with error bars representing the standard deviations. The data were statistically processed using Statistica software (StatSoft Inc., version 12, Tulsa, OK, USA). Remote and outlier values were subtracted based on box graph visualization. The statistical significance against the control was calculated using the non-parametric Wilcoxon matched-pairs test with *p*-values of 0.05 and less.

## 3. Results

### 3.1. Nanoparticle Preparation and Characterization

**Figure 1:**
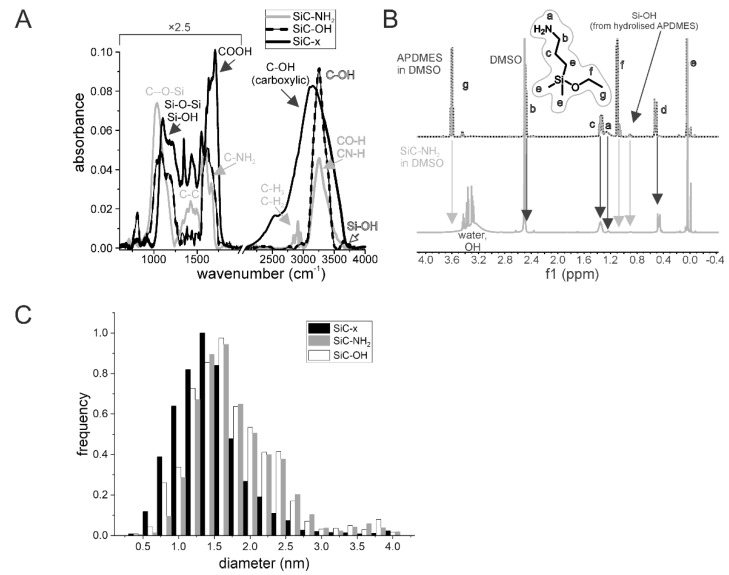
Characterization of the SiC-based NPs. The FTIR spectra (**A**) and size distribution (**C**) of the SiC-based NPs with differing surface terminations based on AFM measurement of individual NPs height. The NMR spectra of APDMES and SiC–NH_2_ (**B**).

The preparation of SiC–x and SiC–OH NPs has been reported previously [32,33,34]. SiC–NH_2_ NPs were prepared from SiC–OH NPs via silanization as described in Materials and Methods. Figure 1A shows the infrared spectra of all the SiC-based NPs. In the case of the SiC–x NPs, the majority of the surface groups are carboxyl groups (COOH). Following the elimination of the carboxyl groups using NaBH_4_, reduction is clearly visible from the disappearance of the peak at 1724 cm^-1^, and the shift and shrinkage of the OH related peak at around 3000 cm^-1^ renders the surface hydroxyl terminated (blue curve—SiC–NH_2_). The presence of 3-aminopropyl(diethoxy)methylsilane (APDMES) used for the preparation of the SiC–NH_2_ enhances the C–O–Si related peak at around 1040 cm^-1^. New peaks appear due to the presence of C-C, C-H and N-H bonds at around 1400–1500, 2800–300, and 1550–1650 cm^-1^, respectively. 

Nuclear magnetic resonance (NMR) also indicated the presence of APDMES (Figure 1C). The disappearance of the detectable ethoxy part indicates that all the APDMES hydrolyzed during the reaction, and the lack of the existence of Si–OH groups indicates that the APDMES is linked to the surface. Hydrolyzed but non-linked APDMES has an Si–OH group that was detectable only in the control, i.e., not in the SiC–NH_2_ NP sample, thus indicating successful surface modification.

Atomic force microscopy (AFM, over 300 particles for each particle type) measurements revealed that the height of SiC-based NPs was under 5 nm (Figure 1B). No significant changes were observed following surface modification, with the exception that the mean size shifted slightly from 1.5 nm to 1.7 and 1.8 nm for the SiC–OH and SiC–NH_2_ respectively, which was caused by a certain degree of aggregation. Nevertheless, the mean sizes of the SiC-based NPs applied in this study were 1.5 nm, 1.7 nm, and 1.8 nm for the SiC–*x*, SiC–OH, and SiC–NH_2_ NPs respectively.

### 3.2. Determination of Zeta Potential in Different Solutions 

**Figure 2:**
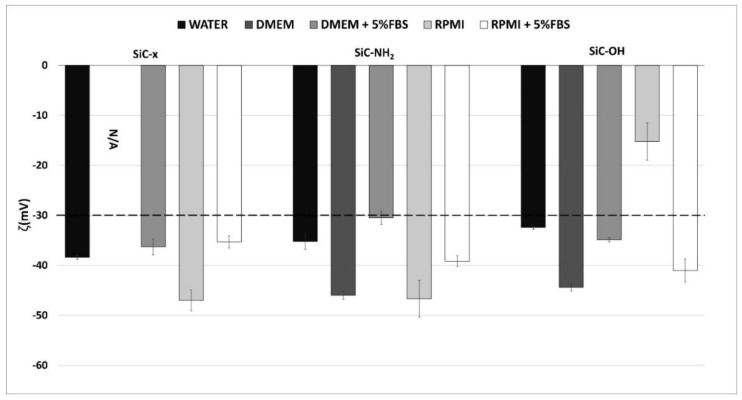
Value of ζ for the SiC-based NPs in different solutions. Parameter ζ was measured on NPs in water, DMEM and RPMI 1640 with and without the addition of 5% FBS.

The behavior of NPs is closely related to their zeta potential (ζ), which reflects the stability of the colloidal suspension of the NPs. By approaching parameter ζ to 0 mV, the colloidal stability worsens, as the NPs attract each other and agglomerate. The NPs used herein were prepared as a stock solution in water and the influence of the biomolecules that originated from the cell cultivation media on the ζ was assessed. Thus, Dulbecco’s modified Eagle medium (DMEM) (experimental cultivation medium for osteoblasts) and RPMI 1640 medium (a standard cultivation medium for monocytes/macrophages) were used as appropriate solutions for the estimation of ζ under biological conditions—in particular cases supplemented with FBS. All the NP types in water exhibited approximately the same ζ, i.e. around −35 mV (Figure 2). The transfer of NPs into the cell cultivation media shifted the ζ to around -45 mV. Parameter ζ decreased only for the SiC–OH NPs in the RPMI medium that evinced into low colloidal stability. The value of ζ of the SiC–x NPs in the DMEM medium is not presented since the SiC–x NPs evinced such a high surface charge (ζ of higher than ±50mV) that they could not be centrifuged out and analyzed. The media supplemented with 5% FBS resulted in a shift in the ζ back to approximately -30 mV, which is considered to be the colloidal stability level [35,36].

### 3.3. Metabolic Activity of the Different Cells Following NP Treatment

The metabolic activity of the different cells (human osteoblasts—SAOS-2; and monocyte/macrophages—THP-1) following their exposure to SiC-based NPs was measured using the MTS test under standard (5% FBS in the medium from the outset) and non-standard (no FBS in the medium for the first 6 h followed by incubation in the medium supplemented with 5% FBS) conditions.

**Figure 3:**
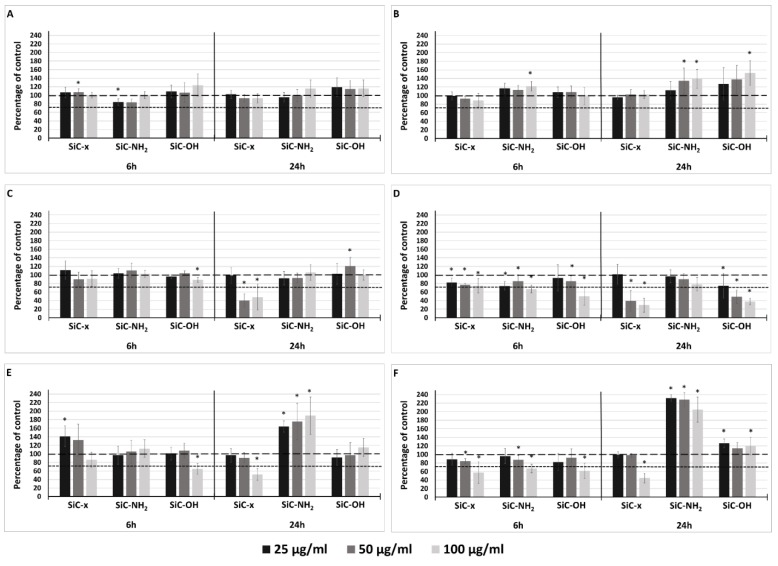
Metabolic activity of human cells treated with SiC-based NPs. Osteoblasts (SAOS-2) (**A**,**B**), adherent THP-1 (macrophage-like cells) (**C**,**D**), and suspension THP-1 (monocytic cells) (**E**,**F**) under standard cultivation conditions (**A**,**C**,**E**) and non-standard cultivation conditions (**B**,**D**,**F**). The data is presented in the form of means, deviation marks represent the standard deviation, the star symbol (*) denotes statistically significant changes against the control levels (Wilcoxon matched-pairs test, *p* < 0.05). The long dashed line shows 100% of the untreated control, the short dashed line shows 75% of the untreated control (cytotoxic level).

Figure 3A shows that the metabolic activity of a well-described and established osteoblastic cell line under standard conditions was not affected by any of the concentrations and types of NPs used. The metabolic activity was approximately at the same level as the control (untreated) cells in all cases. In the case of non-standard conditions (Figure 3B), the metabolic activity after 6 h was comparable to that under standard cultivation conditions. However, after 24 h of incubation, the cellular metabolic activity of the SiC–NH_2_ and SiC–OH NPs at higher concentrations increased significantly. 

The human monocytic cell line—THP-1—was cultivated in two forms, i.e. as a suspension of monocytes and as substrate-adherent macrophage-like cells. Both these types of immune cells were tested for their metabolic activity following the administration of the differing SiC-based NPs. 

In the case of tissue-specific, macrophage-like cells, the obvious effect of FBS medium supplementation was observed. Within the first 6 h of incubation under standard conditions, none of the NPs (with PCs) significantly affected the cellular metabolic activity (Figure 3C). A visible cytotoxic effect (metabolic activity under 75% of the control [37]) was observed later (after 24 h of incubation) with respect only to the two highest SiC–x NP concentrations. Under non-standard conditions (bare NPs), the metabolic activity was significantly reduced after 6 h by all the NP types and concentrations (Figure 3D). Moreover, all the NPs had attained the cytotoxic level at this time point at the highest concentration applied. After 24 h of incubation, substantial differences were observed between the differing NP types. After 6 h, the negative impact of the SiC–NH_2_ NPs on the cells was insignificant; these NPs appeared to be harmless. Conversely, the SiC–x and SiC–OH NPs were significantly cytotoxic with the effect being dose-dependent. 

With respect to the monocytes under the standard cultivation conditions (Figure 3E), after 6 h most of the NPs had not induced any changes in the cell metabolic activity, with two exceptions. The SiC–OH NPs at the highest concentration reduced the metabolic activity to the cytotoxic level and the SiC–x NPs at the lowest concentration significantly increased the metabolic activity of the monocytes. After 24 h, the SiC–x NPs at the highest concentration reduced the monocytic metabolic activity to the cytotoxic level. The metabolic activity of the cells treated with SiC–OH NPs remained at or returned to the level of the untreated control cells. However, the SiC–NH_2_ NPs markedly increased the metabolic activity of the monocytes (to around 200% of the control) after 24 h. Under non-standard conditions (Figure 3F), the metabolic activity was slightly reduced in all the cases after 6 h; however, after 24 h the situation was similar to that of the standard cultivation conditions. Again, the only cytotoxic NPs consisted of the SiC–x NPs at the highest concentration applied, whereas the SiC–OH NPs were at the level of the control cells and the SiC–NH_2_ treated cells again, surprisingly, doubled their metabolic activity compared to the control. 

The tested NPs induced such significant differences in the metabolic activity of the suspension monocytes that further experiments were initiated focusing on these cells as treated with the highest applied NP concentration under non-standard cultivation conditions for 24 h.

### 3.4. Cell Number, Mitochondrial Mass, and Mitochondrial Potential

**Figure 4:**
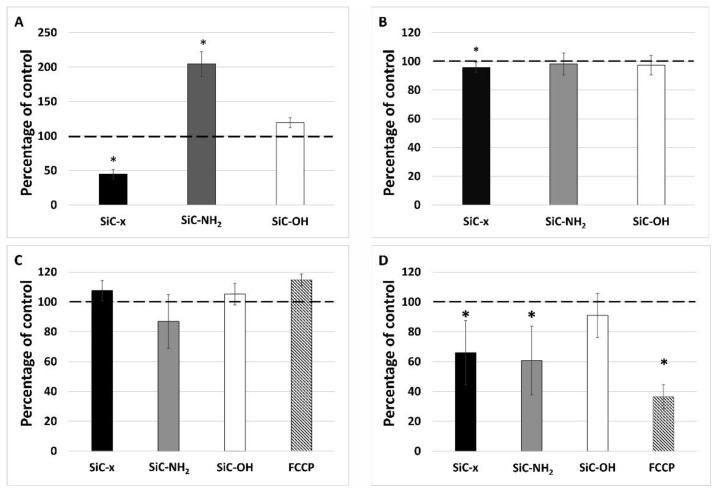
Metabolic activity (**A**), cell number (**B**), mitochondrial mass (**C**), and mitochondrial potential (**D**) after the 24-hour treatment of monocytes with differing SiC-based NPs. The data is presented in the form of means, deviation marks representing the standard deviation, the star symbol (*) denotes statistically significant changes against the control levels (Wilcoxon matched-pairs test, *p* < 0.05). The dashed line shows 100% of the untreated control.

On that account these monocytes were further characterized with respect to the cell number (Figure 4B), mitochondrial mass (Figure 4C) and mitochondrial potential (Figure 4D) because each of them can influence the metabolic activity determined by used method (MTS assay). This method is based on the conversion of a tetrazolium compound to a colored formazan product which is presumably accomplished by NADPH or NADH produced by dehydrogenase enzymes in metabolically active cells. These dehydrogenases are located in mitochondria as well as in cytosol. In spite of their differing metabolic activities (Figure 4A), the number of monocytes was comparable following treatment with differently-terminated SiC-based NPs (Figure 4B). Even though the SiC–x NP treated cells demonstrated a statistically significant decrease in their number (to 95% of the control cells), it did not reflect the significant decrease in their metabolic activity (to 50% of the control cells) (Figure 4A). 

In order to monitor the role of mitochondria in the increased metabolic activity, the overall mitochondrial mass and membrane potential were evaluated using flow cytometry. Figure 4C shows no significant differences in the mitochondrial mass (determined using a fluorescent probe independent of the mitochondrial potential) observed for the cells treated with all three types of NPs tested over 24 h. The determination of the mitochondrial membrane potential was conducted in the same manner as that of the detection of the mitochondrial mass; however, the probe accumulation in mitochondria is highly dependent on their membrane potential as confirmed via FCCP, which was used as an uncoupling agent. As shown in Figure 4D, the SiC–x NPs significantly decreased the mitochondrial potential of the monocytes. Surprisingly, the same phenomenon was also observed in the cells incubated with SiC–NH_2_ particles whose metabolic activity (Figure 4A) was particularly high. The SiC–OH NP treated cells evinced a non-significant decrease in their mitochondrial potential that was comparable to the untreated control. 

### 3.5. Cytokine Release from the Monocytes Following Treatment with Differing SiC-Based NPs 

**Figure 5:**
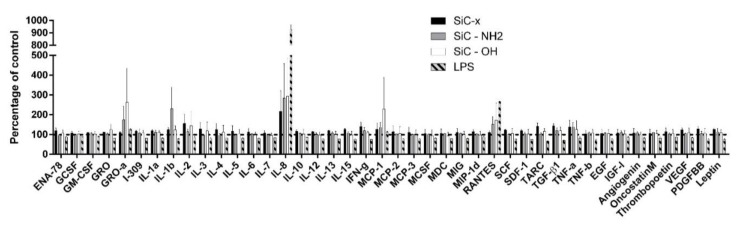
Cytokines detected in the supernatant from the suspension THP-1 monocytes. Cells were treated with 100 μg/mL of SiC-based NPs or LPS under non-standard conditions. After 24 h of incubation, cell supernatant was harvested and analyzed for the presence of a broad panel of cytokines. The data was normalized and is presented as a percentage of the untreated control supernatant levels. The error bars show standard deviation. The dashed line shows 100% of the untreated control.

Cytokines play a key role in cell signaling and this role is even more important in the immune system in terms of mediating its response to different irritating agents. A broad panel of 42 cytokines was tested in order to determine the most important molecules related to the effect of SiC-based NPs on monocytes. Following normalization to the NP untreated control, various cytokine profiles induced by differently-terminated SiC-based NPs were detected (Figure 5). The release of all the detected cytokine types from the cells treated with SiC–x NPs was generally lower than those treated with SiC–NH_2_ and SiC–OH NPs. The pro-inflammatory cytokine interleukin 8 (IL-8) was the only cytokine released at a significant level by the SiC–x NP stimulated cells. High levels of IL-8 were also detected in the cells treated with other types of NPs. Moreover, treatment with SiC–NH_2_ and SiC–OH NPs induced a significant increase in a variety of cytokines such as pro-inflammatory MCP-1, RANTES, and IL-1β and pro-mitogenic GRO-α compared to the untreated control and the cells treated with SiC–x NPs. All of these cytokines were also produced by the positive control cells (cells treated with lipopolysaccharide—LPS), but at an elevated level. 

### 3.6. Morphological Changes in the Cells Following Long-Term Treatment with SiC-Based NPs

**Figure 6:**
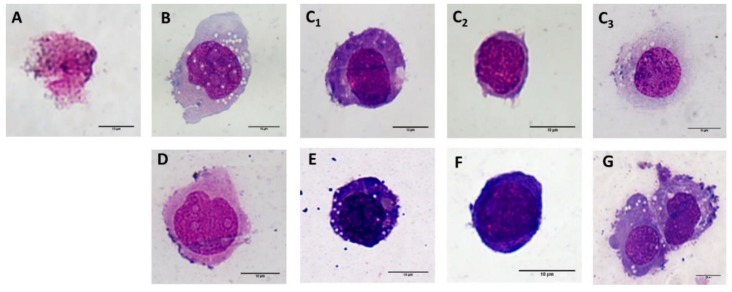
Microscopic analysis of the morphological changes on the THP-1. Representative images of the monocytic THP-1 cells after 7 days of incubation with 100 μg/mL SiC–x NPs (**A**), SiC–NH_2_ (**B**) and SiC–OH (**C_1_**—dendritic cell-like; **C_2_**—monocytic; and **C_3_**—macrophage-like). Control cells (monocytes, incubated in the same manner as the NP-treated cells (10,000 cells/cm^2^) (**D**), chemically-differentiated monocytes to dendritic-like cell morphology (**E**), monocytes (kept in the culture – 133 000 cells/cm^2^) (**F**), and chemically-differentiated macrophage-like cells (PMA treatment) (**G**). All the images were captured at 100× magnification; the scale bar represents 10 µm.

Since no change was observed in the monocyte morphology following short-term incubation (24 h) with NPs of differing surfaces, the long term (7 days) influence of the various SiC-based NPs (100µg/mL) on the monocytic cells was determined; Figure 6 shows the representative morphology of the most commonly-found cells. The control monocytes (Figure 6D) were treated in the same way as the other NP-treated cells (6 h incubation in FBS-free medium, the rest of the incubation time in the medium supplemented with 5% FBS). After 7 days, the cells demonstrated only slight changes in the cytoplasm content compared to fresh THP-1 cells sub-cultured in the standard way (Figure 6F). More dramatic changes were observed in all of the NP-treated cells. All of the SiC–x NP treated cells were found to be dead and only cell debris could be observed (Figure 6A). Treatment with SiC–NH_2_ (Figure 6B) induced an increase in the total number of cells (data not shown) compared to all the other samples, and a significant number of adhered cells with a macrophage-like morphology similar to the cells that were chemically-differentiated into macrophage-like cells (Figure 6G). However, a high number of cells remained in suspension with a morphology similar to the control monocytes (Figure 6D). An interesting mixture of morphology types was observed with respect to SiC–OH treatment (Figure 6C_1-3_). While a portion of the cells revealed a macrophage-like morphology (Figure 6C_3_) as in the SiC–NH_2_ treated cells (Figure 6B), some of the cells also acquired a dendritic cell-like morphology (Figure 6C_1_) similar to the cells that chemically-differentiated into dendritic-like cells (Figure 6E). The remaining cells were either dead (only a small number) or resembled fresh THP-1 cells (Figure 6C_2_).

## 4. Discussion

It is known that the majority of particles tend to change their ζ following the transition into biological fluids [38,39]. Even though amine-terminated surfaces usually have positive zeta potential in salt-free solutions and acidic pH, the data presented herein showed highly negative ζ in case of SiC–NH_2_ NPs. This could be explained by incomplete functionalization of the NP surface. SiC-based NPs are rich in surface groups as one can see in the FTIR spectra (Figure 1A). The overall zeta potential is determined by the salinity and counterions in the solution, the pH, and the surface chemistry. Among the amine groups, SiC–NH_2_ NPs surface likely contains unreacted hydroxyl and silanol groups, and C=C double bonds. This influences the surface potential because of the small distance between particular hydroxyl groups [33], sterical hindrance is expected. The SiC-based NPs presented herein proved to be fully dispersed both in water and in the cultivation medium even when supplemented with FBS, which suggests that the particles remained in the colloid and thus were able to interact with the cells as individual entities. However, a negative ζ may prevent direct contact with a negatively-charged cellular membrane. Nevertheless, the effect induced by the SiC-based NPs on the various cells is apparent, thus indicating the existence of interaction. Moreover, other research has pointed out the potential for NP-cell interaction under such conditions [40,41,42]. The only exception where the ζ was shifted to a surface charge range that favored cluster formation involved the SiC–OH particles incubated in the RPMI 1640 medium. This shift was not observed for the DMEM medium and, from the simple comparison of the media composition, it was not possible to determine a clear link. Some ingredients were presented at strikingly different concentrations (e.g., sodium phosphate monobasic in the RPMI compared to the DMEM (800 mg/l and 125 mg/l respectively)), which may have been the reason for such a change. The dramatic drop in the ζ potential of the SiC–OH NPs (to -17 mV) in the RPMI medium was restored to the ζ corresponding to the stable colloidal solution when the NPs were incubated in the RPMI medium supplemented with FBS. Thus, in this case, the FBS inhibited aggregate formation. The aggregation preventive properties of FBS have already been described by Balek et al. concerning nanodiamonds [43]. 

The concentration gradient of the NPs used herein was based on our previous research on different types of ultra-small silicon-based NPs [42,44]. In both of these studies, said concentration gradients proved to have an impact on metabolic activity of osteoblastic cell line (SAOS-2). Surprisingly, the results showed that irrespective of the NP surface termination, concentration, and cultivation conditions, none of the SiC-based NPs exerted a significant effect on the metabolic activity of these cells in the short-term experiment (24 h). In context of further results presented herein, it seems that used doses of NPs are inert in respect of osteoblasts but cause response in much more responsive cells such as immune cells. Furthermore, the slight elevation in the metabolic activity of the osteoblasts treated with SiC–NH_2_ and SiC–OH after 24 h of incubation indicated possible cell–NP interactions with potentially longer manifestation times. 

The same NPs were also tested with respect to tissue-specific immune cells (adherent THP-1 cells, sometimes referred to as macrophage-like cells). In this case, the initial FBS supplementation of the cultivation medium and, thus, the immediate formation of PCs on the NPs, proved to be the overall key factor that protected the cells from an NP-induced decrease in metabolic activity. This finding corresponds with the previously reported cell-protective impact of protein adsorption onto NPs [20,21,29]. However, this protective effect was not sufficient to prevent a decrease in the metabolic activity of the cells treated with the two highest SiC–x NP concentrations after 24 h. Under non-standard cultivation conditions, the bare NPs (without PCs) initiated a significant decrease in the metabolic activity of these cells after a short incubation period. However, following the removal of the medium containing the NPs and its replacement with a fresh FBS-containing medium (at the 6 h time point), the effect of the SiC–NH_2_ NPs was neutralized and the cell activity was restored to the level of the untreated cells. A similar protective effect of PC was observed in the cells treated with the lowest concentration of SiC–x NPs. Moreover, the higher SiC–x NP concentration accentuated the harmful effect of these NPs, which could have meant that the SiC–x NPs induced irreversible harm to the cells within the first 6 h of incubation, while the SiC–NH_2_ NPs were less harmful and may have initiated apoptotic rescue mechanisms [45]. This observed phenomenon implies that a certain critical concentration of SiC–x and SiC–NH_2_ NPs exists and that, within the first 6 h, it exerts a decisive influence on the fate of cells. The same situation did not apply, however, in the case of the SiC–OH NPs, where an ongoing concentration-dependent toxic effect was observed even after the NP-containing medium had been discarded. In this case, the SiC–OH NPs in the RPMI medium without FBS exhibited the ζ of -17 mV, thus rendering them prone to NP aggregation. Such aggregates cannot be dissolved via the simple addition of FBS and, moreover, are prone to sedimentation. These aggregated sediments cannot easily be discarded in the same manner as the non-clustered particles (SiC–x and SiC–NH_2_ NPs) which remained in the colloid. Thus, SiC–OH NPs may exert an ongoing negative impact on cellular metabolism which may be caused, for example, by the simple mechanical blocking of the membrane due to the quantity of NP clusters, thus resulting in the occurrence of harmful changes in the cellular membrane phase (i.e., gelation) [46]. Since the ζ of the SiC–OH NPs in the RPMI medium supplemented with FBS was -41 mV, the NPs most probably remained in the colloid and exerted no impact on the cellular metabolism. The statistical significances are not presented since they were not considered to be biologically relevant.

Furthermore, when the NPs were tested with respect to monocytic cells, the protective properties of FBS supplementation were also observed; however, the surface termination of the NPs proved to be highly important. In this case, slightly different cultivation conditions were applied (the NPs remained in the incubation medium throughout the whole of the experiment) since the cultivation of suspension cells requires a different approach. The SiC–x NPs demonstrated a similar effect on cellular metabolism under both standard and non-standard conditions. The SiC–NH_2_ NPs proved to be harmless under standard conditions (FBS supplemented) within the first 6 h of incubation and, interestingly, longer cultivation times led to a significant increase in the metabolic activity. Under non-standard conditions, the observed impact of the SiC–NH_2_ NPs on the cellular metabolism was similar to that seen previously with the macrophage-like THP-1. The potential rescue from apoptosis discussed previously concerning these particles manifested itself to a remarkable extent. The SiC–OH NPs exhibited strong FBS-supplementation dependence with regard to their cytotoxic abilities. Under standard cultivation conditions, the monocytic cells responded in the same manner as macrophage-like cells, the explanation for which could be the same as for the previously-described macrophage-like cells, despite monocytic THP-1 cells being non-adherent. As such, the monocytes may have come into contact with the clustered SiC–OH NPs only within the first 6 h since the plate was incubated on a shaker so as to prevent sedimentation. After this time point, the shaker was switched off and, most probably, the clusters so formed sedimented, while the cells remained in suspension and subsequently encountered the NPs. The 24-hour time point confirmed the enormous impact of surface termination on the NP-cell interaction as previously reported [47,48]. This phenomenon was clearly observed particularly under non-standard conditions with the highest concentration of NPs applied.

The MTS assay employed is commonly considered a reflection of the cellular mitochondria function due to the principle thereof (the reduction of the tetrazolium compound into formazan via cellular NAD(P)H dehydrogenase), i.e., the dehydrogenases are most commonly found in the mitochondria. Even though the results of the MTS test suggested that the cellular metabolism differs significantly depending on the type of NP, the other results obtained demonstrated that after 24 h of incubation, the cells treated with all three types of NPs exhibited approximately the same mitochondrial mass. Significant and unexpected changes were, however, identified with respect to the mitochondrial potential. The SiC–NH_2_ NP-treated cells evinced approximately the same mitochondrial potential as the SiC–x NP-treated cells with completely the opposite metabolic activity detected. As some researchers have pointed out, while the MTS and MTT assays do not necessarily exclusively reflect the mitochondrial NAD(P)H dehydrogenase activity, the other dehydrogenases participate in the chemical reaction to a far lower extent [49,50]. This could indicate that the SiC–NH_2_ NP treatment led to the switching of the cellular metabolism, most probably from oxidative phosphorylation to glycolysis, concerning which glyceraldehyde 3-phosphate dehydrogenase may have performed the reduction of tetrazolium salt observed via the MTS assay. This switch may, in turn, indicate that the SiC–NH_2_-stimulated monocytes were directed towards differentiation into activated M1 macrophages, which are known to produce ATP primarily from glycolysis since oxidative phosphorylation is used as an ROS producer to efficiently kill bacteria [51,52].

Since different reactions were observed between the monocytic cells and the various SiC-based NPs tested, it was clear that their reaction with respect to cytokine release should be examined. There is no information in the literature regarding cytokine production following cell stimulation with ultra-small SiC-based NPs, therefore a broader overview was considered necessary. Thus, a panel of 42 cytokines was tested in order to simply and qualitatively determine the presence of certain cytokines. It is common for any foreign material to induce an inflammatory response from immune cells since the surface identity does not usually resemble any naturally-occurring component. A number of studies have reported the production of inflammatory cytokines such as IL-6, IL-1β, and TNF-α related to the nanoparticle treatment of cells [53,54,55]. The results demonstrated that while all the tested SiC-based NPs induced inflammation to a certain extent, the effect was not as strong as in the positive control (LPS treatment). The observed inflammatory pattern suggests that monocytic THP-1 cells respond and interact with all types of SiC-based NPs. Moreover, the production of classical pro-inflammatory cytokines, such as IL-1β and TNF-α, supports the potential for M1 macrophage differentiation and polarization since such cytokines are commonly connected to these cells. In addition, the other cytokine species observed at higher levels such as RANTES, IL-8, and MCP-1 indicated that NPs activate monocytes and act as pro-inflammatory agents. Particularly high levels of IL-8 were observed, thus suggesting that these NPs could potentially lead to a granulocytic immune cell response which is known to be one of their most common functions in vivo. IL-8 has also been demonstrated to act as an angiogenesis mediator by inducing chemotactic and proliferative activity in endothelial cells [56]. The presence of IL-8 and MCP-1, however, should be of concern since the combination of these two cytokines is known to attract neutrophils and to promote inflammation. Fortunately, no allergic response (increased levels of IL 4, 5, and 13) was detected via the application of the tested NPs; their presence would have prevented the future use of such NPs in vivo since a strong allergic reaction could result in fatal consequences (e.g., anaphylactic shock in living organisms). Increased attention is being devoted to the role of NPs in the development of new allergies, which is increasingly becoming a subject of concern in human medicine [26,57]. The non-presence of pro-allergic cytokines produced by monocytic THP-1 cells in response to SiC-based NP treatment is thus desirable and indicates that this nanomaterial is harmless in this respect; however, significant concerns with respect to acute inflammation have been raised by high levels of IL-8 and MCP-1. These cytokines also present promising application potential concerning their positive influence on neutrophils. The fact that monocytic cells interact with nanoparticles, while not becoming over-activated, identifies these SiC-based NPs as a potential immunomodulatory material. 

Practically no long-term studies of the influence and differentiation abilities of NPs have been conducted to date. *In vivo*, monocytes possess the ability to differentiate into macrophages or dendritic cells dependent on the needs of the immune system. Since various pro-inflammatory cytokines were detected after 24 h of incubation with the particles, the possible impact on changes in the cellular morphology were studied after a longer period (7 days). The cells cultured with SiC–NH_2_ and SiC–OH NPs evinced significant changes in their morphology, thus suggesting the application potential of these NPs as immunomodulatory material. Since most of the cells treated with SiC–NH_2_ particles were transformed into adherent macrophage-like cells and the SiC–OH NPs provided cells that differentiated into macrophage-like and dendritic cell–like morphologies, both of these particles might potentially be employed as ex vivo stimuli for immune cell therapy via long-term treatment as well as for the stimulation (again, ex vivo) of the immune response in immunodeficient individuals. This ex vivo application has been proposed with respect to various silicon-based NPs [54,58]. However, in the case of the SiC–NH_2_ NPs, further attention should be devoted to the observed increase in the cell count (not published), which could indicate the mean overstimulation of the cells and could possibly lead to an undesirable excessive response. Consequently, SiC–OH NPs might be considered eligible for further ex vivo application as suggested since, although the number of cells did not differ from the control, differentiation was present. Morphological changes were also observed in the control cells that were treated in the same manner as the NP-treated samples. While the cells remained in suspension, they evinced a markedly higher amount of cytoplasm. This change was most probably caused by the suboptimal cultivation conditions (i.e., low cell density) that were applied so as to retain the same conditions as those of the NP-treated cells. Another possible explanation is that standard cultivation (high cell density) was conducted in the medium supplemented with heat inactivated FBS (i.e., inactive complement), while the particle-related experiments (cultivation in low cell density) were conducted in the medium with FBS without heat inactivation (i.e., active complement).

All the aforementioned potential applications must, however, be further tested on primary cells from human donors. The ex vivo stimulation of cells and the subsequent return of such cells into the body presents a further direction for immunomodulation research. Cells stimulated in this way could produce cytokines that positively influence the number and activity of neutrophils, which offers potential with concern to neutrophil-related immunodeficiencies [59].

## 5. Conclusions

Even though different termination of SiC-based NPs does not have a significant effect on their colloidal stability in the cultivation media, it plays a key role in respect to human cells. Monocytes, being a key part of the immune system, produce a variety of cytokines upon contact with these NPs and mark them as a potential immunomodulatory material. Further research regarding changes in metabolic pathways, differentiation capabilities and cytokine productions were however of major interest in order to correctly establish the application potential of these NPs.
